# Antibodies Targeted to the Brain with Image-Guided Focused Ultrasound Reduces Amyloid-β Plaque Load in the TgCRND8 Mouse Model of Alzheimer's Disease

**DOI:** 10.1371/journal.pone.0010549

**Published:** 2010-05-11

**Authors:** Jessica F. Jordão, Carlos A. Ayala-Grosso, Kelly Markham, Yuexi Huang, Rajiv Chopra, JoAnne McLaurin, Kullervo Hynynen, Isabelle Aubert

**Affiliations:** 1 Brain Sciences, Sunnybrook Research Institute, Toronto, Ontario, Canada; 2 Imaging, Sunnybrook Research Institute, Toronto, Ontario, Canada; 3 Department of Laboratory Medicine and Pathobiology, Faculty of Medicine, University of Toronto, Toronto, Ontario, Canada; 4 Department of Medical Biophysics, Faculty of Medicine, University of Toronto, Toronto, Ontario, Canada; 5 Centre for Research in Neurodegenerative Diseases, University of Toronto, Toronto, Ontario, Canada; 6 Unidad de Biología Molecular, Facultad de Farmacia, Universidad Central de Venezuela, Los Chaguaramos, Caracas, Venezuela; Massachusetts General Hospital and Harvard Medical School, United States of America

## Abstract

Immunotherapy for Alzheimer's disease (AD) relies on antibodies directed against toxic amyloid-beta peptide (Aβ), which circulate in the bloodstream and remove Aβ from the brain [1], [2]. In mouse models of AD, the administration of anti-Aβ antibodies directly into the brain, in comparison to the bloodstream, was shown to be more efficient at reducing Aβ plaque pathology [3], [4]. Therefore, delivering anti-Aβ antibodies to the brain of AD patients may also improve treatment efficiency. Transcranial focused ultrasound (FUS) is known to transiently-enhance the permeability of the blood-brain barrier (BBB) [5], allowing intravenously administered therapeutics to enter the brain [6]–[8]. Our goal was to establish that anti-Aβ antibodies delivered to the brain using magnetic resonance imaging-guided FUS (MRIgFUS) [9] can reduce plaque pathology. To test this, TgCRND8 mice [10] received intravenous injections of MRI and FUS contrast agents, as well as anti-Aβ antibody, BAM-10. MRIgFUS was then applied transcranially. Within minutes, the MRI contrast agent entered the brain, and BAM-10 was later found bound to Aβ plaques in targeted cortical areas. Four days post-treatment, Aβ pathology was significantly reduced in TgCRND8 mice. In conclusion, this is the first report to demonstrate that MRIgFUS delivery of anti-Aβ antibodies provides the combined advantages of using a low dose of antibody and rapidly reducing plaque pathology.

## Introduction

Evidence of Aβ toxicity, including in the brain of people with AD [11], reinforces the need to improve current anti-Aβ treatment. Current treatments in AD patients require the long-term administration of high doses of antibodies against Aβ in the bloodstream in order to remove Aβ plaques from the brain of AD patients [12], [13]. In mouse models of AD, cognitive improvement following immunotherapy was obtained with intravenous or intraperitoneal administration of high doses of 500 µg of anti-Aβ antibodies [14]–[16]. Considering that only up to 0.1% of anti-Aβ antibodies administered peripherally can reach the brain [17], most, administered antibody remains in the bloodstream. Recently, it was shown in transgenic mice that a low dose of anti-Aβ antibodies administered directly into the brain was more efficient at clearing Aβ than peripheral injections of high doses of antibodies, with a concomitant reduction in vascular Aβ-pathology [3]. Targeted delivery of antibodies in the brain allows for a greater proportion of anti-Aβ antibodies to reach the affected brain region, which may result in better treatment efficacy in AD patients.

Direct delivery of anti-Aβ antibodies into the brain of animal models by intracranial injection [18] or skull-cap removal [4] is known to reduce plaque load within 3 days, however the invasive nature of these procedures would raise serious safety concerns in humans. Alternatively, transcranial MRIgFUS is a relatively non-invasive approach of delivering therapeutics to the brain. It has been shown to transiently enhance the permeability of the blood-brain barrier (BBB) [5] and allow for the delivery of therapeutic agents, as large as antibodies, into the brain of animals [6], [7].

Previous studies investigating the mechanisms of MRIgFUS delivery have reported localized transport of agents across the BBB by transcytosis and the widening of tight junctions and channels [19], [20]. These effects on the BBB were reversible when FUS was maintained at a pressure amplitude of 0.4 MPa or less [21]. Between 4–6 hours following treatment, the integrity of the BBB was restored and no cellular or neuronal damage was detected.

This report assessed the delivery of anti-Aβ antibodies by MRIgFUS and the efficacy of this treatment to reduce Aβ plaque pathology. Specifically, our main objectives were to: 1) rapidly deliver anti-Aβ antibodies to the brain using MRIgFUS and a low (40 µg) intravenously-injected dose of anti-Aβ antibody, and 2) evaluate whether this treatment reduces Aβ plaque load in TgCRND8 mice within a short time-frame of 4 days.

## Methods

### Transgenic mice

TgCRND8 mice encode a double mutant form of amyloid precursor protein 695 (KM670/671NL/V717F) under the control of the PrP gene promoter [10]. TgCRND8 mice develop amyloid pathology by 3 months of age which is accompanied by cognitive deficits [10], [22]. These mice have been well characterized in various immunization paradigms [23], [24]. Male and female TgCRND8 mice [10] of 132–137 days in age were used in this study, an age when only compact plaques are found in the cortex [10].

These studies were conducted with the approval of the Animal Care Committee of Sunnybrook Health Sciences Centre and in compliance with the guidelines established by the Canadian Council on Animal Care and the Animals for Research Act of Ontario.

### MRI-guided FUS delivery

**Figure 1:**
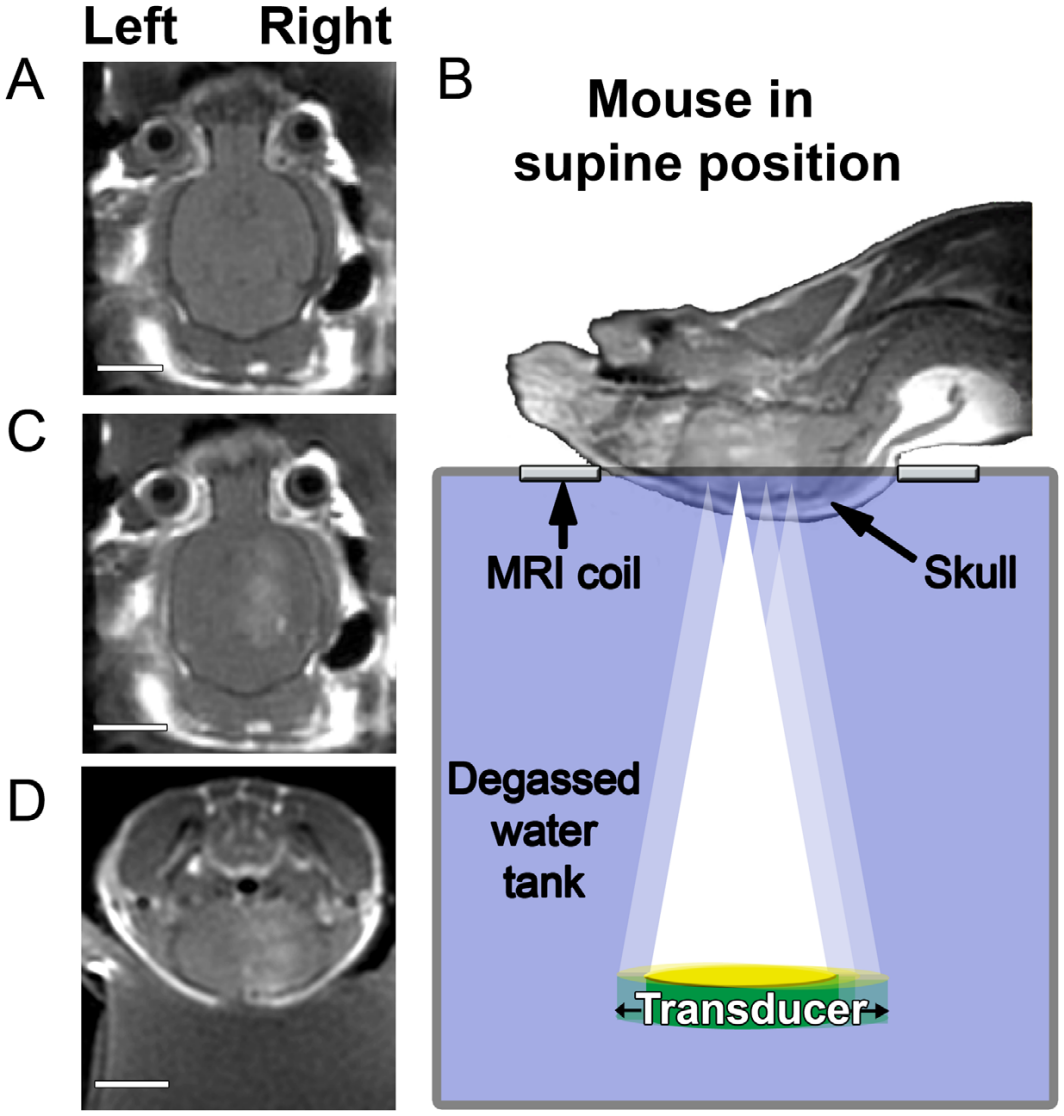
Magnetic resonance imaging-guided focused ultrasound (MRIgFUS) increases the permeability of the blood brain barrier (BBB). T1-weighted contrast-enhanced MRI scans were used to position ultrasound foci prior to treatment (**A**) and following transcranial FUS (**B–D**), were used to monitor the entry of gadolinium in the brain. Mice were positioned in a supine position (**B**) and injected in the tail vein with microbubbles and gadolinium while ultrasound was applied to 4 aligned spots on the right hemisphere of the brain. Increased BBB permeability was monitored by MRI, visualizing contrast enhancement by the influx of gadolinium (**B–D**). [A–D: 128×128, TE/TR = 10.4/500.0, FOV = 4cm, Slice 1mm, ETL = 4, 3NEX]. Scale bars: A, C, D = 0.5 cm.

Mice were anaesthetized with ketamine (150 mg/kg) and xylazine (10 mg/kg). An angio-catheter was inserted into the tail vein and the mouse secured in a supine position (**Fig. 1B**). The FUS system was placed inside a 3.0T MRI for positioning of the target locations using an RF coil with inner dimensions of 3.2 cm by 4.2 cm [9]. A FUS beam was generated from a piezoelectric transducer (10 cm diameter, 8 cm radius of curvature, 0.558 MHz resonant frequency) positioned inside a degassed water tank using an MRI-compatible three-axis motorized system [9]. Four spots, 1.5 mm apart, along the right hemisphere were targeted with ultrasound (0.3 MPa, 120 s, 10 ms bursts/Hz). *Definity* microbubbles (160µl/kg; Bristol Meyers Squibb; mean size 1–2 µm), MRI contrast agent Gadovist (0.1 ml/kg, Schering AG, Berlin, Germany) and 40 µg of BAM-10 antibody (A5213, Sigma Aldrich, St Louis, MO, USA) were delivered through the tail vein at the time ultrasound was applied. BAM-10 is directed against the N-terminus of Aβ (residues 1–10), which represents the optimal target for Aβ clearance [25]. BAM-10 has been shown to be effective at dissociating Aβ aggregates [26] and rescuing cognitive function [4] in mouse models of AD. MRI images were captured to determine the extent of BBB disruption based on the increase of signal intensity from the contrast-enhancement.

### Biotinylated BAM-10 immunoprecipitation analysis

BAM-10 antibody (A3981, Sigma, Aldrich, St Louis, MO, USA) was biotinylated using sulfo-NHS-LC-biotin kit (Pierce, Rockford, IL, USA). Mice treated with MRIgFUS-delivered biotinylated BAM-10 (as described above), were deeply anaesthetized with ketamine (250 mg/kg) and xylazine (17 mg/kg) for intracardial perfusion with saline. Brains were removed and regions from the right and left hemispheres were dissected. Tissue from each hemisphere was homogenized and equalized to 2.5 mg/ml. Lysates (200 µl) were incubated with 20 µl of protein A/G beads (SC-2003, Santa Cruz Biotechnology Inc., Santa Cruz, CA, USA) at 4°C overnight. Antibody was eluted using 20 µl of 2× Laemli sample buffer and boiled for 10 min at 95°C. Samples were run in 10% SDS-PAGE and transferred to nitrocellulose membrane. After blocking with 5% milk in TBS-T membranes were incubated with peroxidase conjugated streptavidin (1∶2,000; Jackson Immuno Research Laboratories, Inc., West Grove, PA, USA) and visualized with chemiluminescence using ECL.

### Co-localization time course of BAM-10 and plaques using immunofluoroscent staining

Sections from mice treated with biotinylated BAM-10 and FUS were incubated with a primary antibody against Aβ (rabbit polyclonal Aβ42; 1∶100; Millipore Corp., Temecula, CA, USA) followed with donkey anti-rabbit Cy3 antibody to reveal Aβ (1∶100; Jackson Immuno Research Laboratories, Inc., West Grove, PA, USA) and streptavidin-Alexa 488 to reveal biotinylated BAM-10. Fluorescent labeling was detected by confocal microscopy (Zeiss Axiovert 100M, LSM 510; Carl Zeiss, Don Mills, Canada).

### Detection of biotinylated BAM-10 and Aβ plaques

Detection of the biotinylated BAM-10 antibody was revealed with streptavadin-horseradish peroxidase (Vectastain Elite, Vector Laboratories, Burlingame, CA, USA) followed by 3,3′-diaminobenzidine (DAB) (Sigma, St Louis, MO, USA).

For the immunostaining of plaques with the anti-Aβ antibody 6F3D, sections were pre-treated with 70% formic acid, 3% hydrogen peroxide in 100% methanol, washed and incubated overnight at 4°C with 6F3D (1∶400; Dako North American Inc., Carpinteria, CA, USA). Sections were then incubated with biotinylated donkey anti-mouse IgG secondary antibody (1∶100; Jackson Immuno Research Laboratories, Inc., West Grove, PA, USA). 6F3D-biotin complexes were revealed with streptavadin-horseradish peroxidase (Vectastain Elite, Vector Laboratories, Burlingame, CA, USA) and DAB. Brightfield images were captured with Zeiss Imager M1 microscope coupled to a digital camera, motorized stage, and the Virtual Slice module (MBF Bioscience, Williston, VT, USA).

To ensure that BAM-10 antibody does not prevent the binding of the antibody 6F3D to amyloid plaques, the above immunohistochemistry procedures were carried out with double-staining of streptavadin-Cy2 (1∶200; Jackson Immuno Research Laboratories, Inc., West Grove, PA, USA) to label BAM-10 antibody and donkey anti-mouse Cy3 (1∶100; Jackson Immuno Research Laboratories, Inc., West Grove, PA, USA) to label 6F3D. The staining for both antibodies was visualized by confocal microscopy (Zeiss Axiovert 100M, LSM 510; Carl Zeiss, Don Mills, Canada).

### Stereology

MRI post-treatment scans were used to identify the area of BBB opening (indicated by the increase in intensity where gadolinium entered the brain) and were measured using Matlab software. The averaged measurements were then used to map out equivalent contours using Stereo Investigator (5.05.1 software; MBF Bioscience, Williston, VT, USA) on the right and left primary and secondary motor cortical regions of brain sections stained for plaques with 6F3D, as described above. According to a randomly selected start point (within 1.94 mm anterior and 0.34 mm posterior of Bregma; a region normally abundant with plaques and within the treatment area), plaque quantification for each animal was based on 6-7 sections at an interval of one in 9. Stereology was performed within each contour with a Zeiss Axioplan 2 microscope coupled to a DEI-750 CE video camera (Optronics, Goleta, CA, USA) and a software-driven Ludl X-Y-Z motorized stage (Ludl Electronic products, Hawthorne, NY, USA) for estimation of plaque number (optical fractionator) and size (nucleator probe) 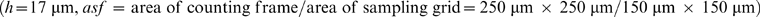.

### Biochemical analysis of Aβ levels

Mice were perfused as described above with saline only and brains were dissected by region. Treated and contralateral areas of the cortex from the left and right hemispheres of all mice were homogenized in a 20 mM Tris-buffered solution containing 0.25 M sucrose, 1 mM EDTA, 1 mM EGTA, and a 1% protease inhibitor cocktail (Calbiochem, La Jolla, CA, USA). The homogenate was separated into two portions for total and soluble Aβ analyses as we have previously published [27]. Soluble Aβ was extracted using DEA/NaCl solution, followed by centrifugation. The supernatant was removed and neutralized for soluble Aβ analysis. The total fraction was solubilized in cold formic acid, followed by sonication and centrifugation. The supernatant was similarly collected and neutralized. Aβ_40_ and Aβ_42_ levels were determined using commercially available ELISA kits (BioSource, Invitrogen, Burlington, ON, Canada). Aβ levels were normalized using total protein concentration as determined using Bio-Rad assays (Bio-Rad Laboratories, Mississauga, ON, Canada).

### Statistical analysis

Statistical analysis was performed using SPSS 17.0 Software. Two-tailed, paired t-tests, α = 0.05, n = 6 for all groups, were used to determine significance between plaque pathology from left versus right hemispheres of the brain for mice in all treatment groups. Analysis of variance (ANOVA) was used to determine the effect on Aβ pathology between treatment groups.

## Results

### Magnetic resonance imaging-guided focused ultrasound (MRIgFUS) locally increases the permeability of the blood brain barrier (BBB)

For antibody delivery through the BBB, three-planar MRI scans were used to position four ultrasound foci along the rostro-caudal axis of the right brain hemisphere of TgCRND8 mice (**Fig. 1A–D**). Mice received a tail vein administration of ultrasound microbubbles (Definity, 160 µl/kg) to disrupt the BBB, and the MRI contrast agent gadolinium (Gadovist, 0.1 ml/kg), at the onset of transcranial MRIgFUS application (0.3 MPa for 120 s, pulsed for 10 ms at 1Hz; below the reported threshold of damage [21]). The entry of gadolinium into the right, targeted hemisphere was observed within 5–10 min following MRIgFUS in the presence and absence of anti-Aβ antibody, BAM-10, confirming a localized disruption of the BBB (**Fig. 1B–D**). When the enhancement seen at the site of BBB disruption was ≤20%, in MRI post-treatment scans acquired 10-15 minutes post-treatment, we found no red blood cells detected in the brain parenchyma using Prussian blue staining (not shown).

### Intravenously injected BAM-10, an anti-Aβ antibody, rapidly entered the brain in areas targeted with MRIgFUS

**Figure 2:**
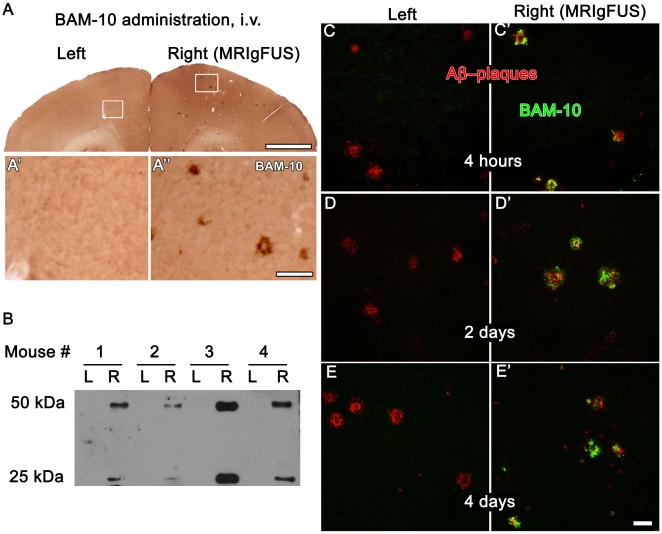
BAM-10 entered the right, MRIgFUS-treated, side of the brain and bound to Aβ plaques. Following intravenous (i.v.) administration of biotinylated BAM-10 and treatment with MRIgFUS, the entry of biotinylated BAM-10 into the brain of TgCRND8 mice was evaluated by processing brain sections with streptavidin-horseradish peroxidase and standard histochemistry procedures (**A**). Biotinylated BAM-10 was not found on left side of the brain (**A′**). In contrast, the right side of the brain, exposed to FUS, had abundant biotinylated BAM-10-deposits (**A″**). Biochemical analysis of brain tissue by immunoprecipitation and western blotting (**B**, 4 mice represented) also showed that biotinylated BAM-10 was not detected on the left (**B**, L) and abundant on the right (**B**, R) side of the brain, which received MRIgFUS. Brain sections from mice sacrificed at 4 hours (**C**, **C′**), 2 days (**D**, **D′**) and 4 days (**E**, **E′**) post-treatment were double stained with streptavidin-Alexa 488 to detect biotinylated BAM-10 (green) and a rabbit Aβ42 antibody conjugated with Cy3 (red). Biotinylated BAM-10 was present at all time points on the right side of the brain receiving MRIgFUS and it co-localized with Aβ-positive plaques. Scale bars: A = 1 mm; A′, A″ = 100 µm; C-E′ = 50 µm.

We then evaluated whether MRIgFUS could deliver a low peripheral dose of BAM-10 into the brain. The same experimental settings, as described above (**Fig. 1B**), were used except BAM-10 was biotinylated to facilitate the downstream detection of BAM-10. At all time points post-MRIgFUS (4 hr, 2 and 4 days), biotinylated-BAM-10 was found bound to Aβ plaques only on the right, MRIgFUS-targeted side of the brain. Localized delivery of biotinylated-BAM-10 to the right hemisphere was demonstrated by immunohistochemistry (**Fig. 2A**), and immunoprecipitation combined with western blotting (**Fig. 2B**). Co-localization of biotinylated-BAM-10 (**Fig. 2C–E′** **, green**) with Aβ plaques, stained with rabbit anti-Aβ42 antibody (**Fig. 2C–E′** **, red**), was confirmed using confocal microscopy. On the right side of the brain, treated with MRIgFUS, all anti-Aβ_42_ antibody positive cortical plaques were also immunopositive for biotinylated-BAM-10, as illustrated in the representative fields of view in Fig. 2. In contrast, on the untreated left hemisphere of the same brain sections, plaques stained only for the rabbit anti-Aβ42 antibody and no biotinylated-BAM-10 was detected. Therefore, in the current conditions, BAM-10 was delivered from the peripheral circulation to the brain only in MRIgFUS targeted areas of the right hemisphere.

### MRIgFUS delivery of BAM-10 to the brain reduces Aβ plaque pathology

**Figure 3:**
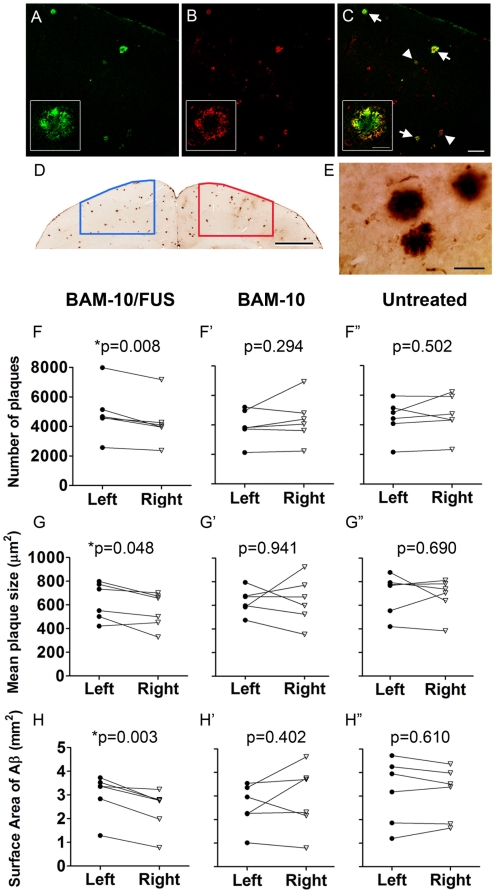
Focused ultrasound delivery of BAM-10 to the brain reduces Aβ plaque pathology in TgCRND8 mice. Coronal sections were stained with streptavadin-Cy2 for (**A**) biotinylated BAM-10 and (**B**) anti-Aβ antibody 6F3D for plaques to demonstrate that 6F3D binds to plaques which are strongly (arrows) and weakly (arrowheads) positive for BAM-10 (**C**). Once it was confirmed that BAM-10 does not interfere with 6F3D plaque detection, sections for mice in each treatment group were stained with 6F3D and the stereology software was used to draw contours outlining the FUS-targeted region (determined from MRI post-treatment scans) on the right side of the brain and an equivalent region on the contralateral side (**D**). Plaques were counted and measured at high magnification (**E**), using stereological methods. In 4 days, the mean (**F**) count, (**G**) size and (**H**) surface area of Aβ plaques on the right, MRIgFUS-targeted side of the brain was consistently reduced in comparison to the left side of the brain only for the BAM-10/FUS-treated mice (**F–H**; n = 6, paired t-tests, p = 0.008, p = 0.048 and p = 0.003, respectively). This difference is unique to BAM-10/FUS treatment as there was no significant difference between right and left side of the brain in mice from other treatment groups (**F′–H′**: BAM-10 treated group, n = 6, paired t-tests, p = 0.294, p = 0.941 and p = 0.402; **F″–H″**: Untreated group, n = 6, paired t-tests, p = 0.502, p = 0.690, p = 0.610). Scale bars: A–C = 100 µm (inset = 20 µm); D = 1 mm; E = 50 µm.

The therapeutic effects of BAM-10 delivery to the brain were evaluated by assessing plaque pathology in cortical areas targeted by MRIgFUS (right hemisphere, presence of BAM-10) to those not targeted by MRIgFUS (left hemisphere, absence of BAM-10). Four month-old TgCRND8 mice were randomly assigned to 4 treatment groups: **BAM-10/FUS** (treated with MRIgFUS and a single 40 µg, i.v. dose of BAM-10), **FUS** (treated with MRIgFUS but no BAM-10), **BAM-10** (40 µg BAM-10 i.v., no MRIgFUS), or **untreated** (no MRIgFUS, no BAM-10). All groups received the same anesthetic procedures. With the exception of the untreated group, all groups were injected with microbubbles and gadolinium in the tail vein. MRIgFUS was applied only to the right hemisphere (as in **Fig. 1**). Four days later, brains were collected and processed for staining with the anti-Aβ antibody 6F3D. To ensure that the delivered BAM-10 antibody did not prevent the binding of 6F3D to Aβ plaques, sections from mice that had been treated with MRIgFUS-delivered biotinylated BAM-10 were stained for plaques with 6F3D (red, **Fig. 3A**) and for detection of BAM-10 antibody with strepavidn-Cy2 (green, **Fig. 3B**). 6F3D was clearly detected on all BAM-10-positive plaques (**Fig. 3C**).

Plaques stained for 6F3D (**Fig. 3D,E**) were quantified in 6–7 sections per animal, using design-based stereology for all groups of mice. Quantification of plaque load was based on Aβ plaque numbers (optical fractionator), cross-sectional area (nucleator) and surface area of Aβ (plaque number X mean cross-sectional area).

Among all groups (untreated, FUS-, BAM-10-, BAM-10/FUS-treated; n = 6 for all groups), statistically significant differences in plaque pathology were found only in BAM-10/FUS-treated mice, comparing the left (untreated) and right (treated) sides of the brain for each mouse (paired t-tests, **Fig. 3F,G,H**). In all mice treated with BAM-10/FUS there were fewer plaques on the right, MRIgFUS-targeted hemisphere (12% reduction), in comparison to their respective contralateral sides (p = 0.008, n = 6, paired t-test, **Fig. 3F**). The mean plaque size was lower on the right hemisphere, in comparison to the left, in 5 out of 6 BAM-10/FUS-treated mice, representing a significant reduction in plaque size (12% mean reduction, **Fig. 3G**). Further, the estimated surface area covered by Aβ plaques was significantly smaller on the right compared to the left hemisphere (p = 0.003, n = 6, paired t-test, **Fig. 3H**) for all mice in the BAM-10/FUS group, with a mean reduction of 23%. Other groups (Fig. 3F′–H′, 3F″–G″), including FUS alone (not shown), had no significant difference in the number of plaques, mean plaque size and surface area covered by Aβ plaques between hemispheres (p>0.05).

### MRIgFUS-delivery of BAM-10 and Aβ levels after 4 days

**Figure 4:**
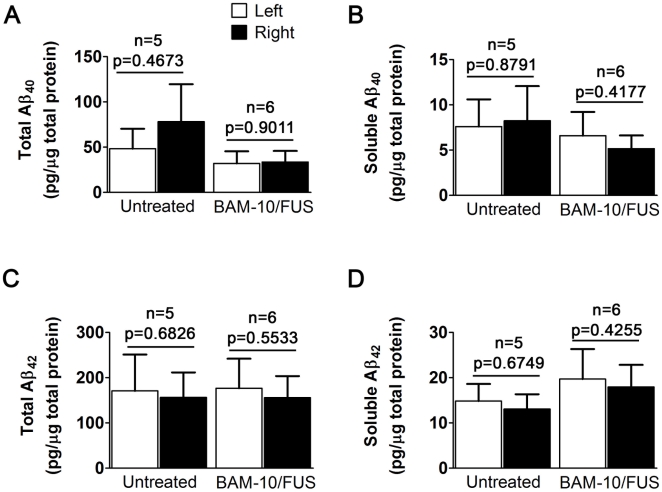
Aβ levels in TgCRND8 mice after one BAM-10/FUS treatment. Cortical homogenates were analyzed for total Aβ_40_ (**A**), soluble Aβ_40_ (**B**), total Aβ_42_ (**C**) and soluble Aβ_42_ (**D**). There was no significant difference detected any Aβ species and fractions between the left and right hemispheres.

Once established that MRIgFUS-delivered BAM-10 could reduce plaque burden after a single treatment, we used Aβ ELISAs to determine Aβ_40_ and Aβ_42_ levels in untreated, BAM-10/FUS-, BAM-10-, and FUS-treated mice. Among all groups, there was no significant difference due to treatment (ANOVA, p>0.05). Paired t-tests indicated no statistically significant left-to-right differences in the levels of Aβ_40_ (total, **Fig. 4A**; soluble, **Fig. 4B**) and Aβ_42_ (total, **Fig. 4C**; soluble **Fig. 4D**) in any of the groups (shown are untreated and BAM-10/FUS).

## Discussion

We demonstrated that MRIgFUS efficiently delivered the anti-Aβ antibody, BAM-10, from the bloodstream to the brain. Once in the brain, BAM-10 bound to Aβ-plaques within hours and remained associated with plaques for at least 4 days. Importantly, we report for the first time that a single MRIgFUS treatment in conjunction with one low dose (40 µg) of intravenous BAM-10 anti-Aβ antibodies reduces the pathology of compact plaques in TgCRND8 mice in terms of the number, mean size and surface area occupied by Aβ. We found no significant difference in total and soluble Aβ loads measured by ELISA. Similar findings have been previously demonstrated in TgCRND8 mice after prolonged active immunization with Aβ_42_ [23]. Similarly, Dodart and colleagues reported changes in plaque levels without changes in Aβ levels in PDAPP mice [28].

At 4 days post-treatment, right cortical areas that received BAM-10 by MRIgFUS delivery had reduced plaque pathology compared to the left side of the brain. Significant reduction of plaque burden was evident based on the number or size of plaques alone (12% reduction in both cases), and the total surface area of Aβ plaques (23% reduction). Considering the variability in the number of plaques in TgCRND8 mice at this age, no significant difference in plaque load was observed between treatment groups. TgCRND8 mice exhibit a very rapid accumulation of Aβ at 4 months of age, resulting in large variances in plaque numbers in all groups of TgCRND8 mice. To evaluate the impact of a single MRIgFUS treatment with BAM-10 on plaque load within each animal, we focused our analyses on pair-wise comparisons of right (treated) and left (untreated) sides of the brain.

The reduction of plaque load in TgCRND8 mice, on the BAM-10/FUS treated hemisphere compared to the untreated side of the brain, was achieved rapidly – within 4 days – and with a single low dose of 40 µg of anti-Aβ antibody. Previous passive immunization studies in AD mouse models, where plaque pathology was reduced [4], [14]–[16], [28], required the systemic administration of anti-Aβ antibodies at concentrations that were 10-fold higher and repeated weekly over several months. Here, the delivery of anti-Aβ antibodies to the brain using non-surgical MRIgFUS yielded a significant reduction in Aβ plaque pathology 4 days after a single treatment. This timeframe of treatment efficacy is comparable to the administration of antibodies directly into the brain using invasive intracranial delivery [4], [18]. Interestingly, in our experimental conditions, we observed that MRIgFUS delivered antibodies to widespread areas of the cortex, thereby targeting all plaques, as illustrated by the co-localization of biotinylated-BAM-10 with Aβ-positive plaques (Fig 2C′–E′).

Using a different mouse model of AD (B6C3-Tg), Raymond *et al.* suggested that anti-Aβ antibodies given intravenously could enter FUS-targeted areas of the brain [8]. We are the first to show that the delivery of anti-Aβ antibodies to the brain using MRIgFUS has a significant therapeutic effect. This represents a substantial advance to the field of MRIgFUS anti-Aβ antibody delivery. We used MRIgFUS, in contrast to FUS alone, to adjust the location of treatment foci and monitor changes in the permeability of the BBB during the experiment. We achieved significant BAM-10 delivery in MRIgFUS targeted brain areas with one BAM-10 dose of 40 µg (i.v.), which is 100 times lower than the dose of anti-Aβ antibody previously used for FUS-delivery to the brain [8]. We have also established the clear co-localization of BAM-10, delivered by MRIgFUS to the brain, with existing Aβ-positive plaques identified with another Aβ-specific antibody. Lastly, we demonstrated MRIgFUS immunotherapy can quickly decrease compact plaque burden after a single treatment in a model of AD with aggressive Aβ pathology.

In conclusion, this is the first report to demonstrate that MRIgFUS delivery of anti-Aβ antibodies rapidly reduces plaque pathology. Immunotherapy treatments showing efficacy within a short time-frame could offer significant clinical benefits to people living with Alzheimer's disease. Furthermore, recent advances in high-field micro-MRI shows promise for detecting Aβ plaques *in vivo* [29]. Eventually, the combination of MRI and FUS could provide the benefit of using micro-MRI to image plaques *in vivo*, and target these areas with FUS-delivered anti-Aβ antibodies. This imaging and delivery system could potentially offer the opportunity for a more tailored therapy and the ability to monitor the efficacy of treatment *in vivo*.
